# Transient Receptor Potential Ankyrin 1 Channel Expression on Peripheral Blood Leukocytes from Rheumatoid Arthritic Patients and Correlation with Pain and Disability

**DOI:** 10.3389/fphar.2017.00053

**Published:** 2017-02-10

**Authors:** Ione Pereira, Saulo J. F. Mendes, Domingos M. S. Pereira, Thayanne F. Muniz, Valderlane L. P. Colares, Cinara R. A. V. Monteiro, Mahiba M. R. de S. Martins, Marcos A. G. Grisotto, Valério Monteiro-Neto, Sílvio G. Monteiro, João B. Calixto, Susan D. Brain, Elizabeth S. Fernandes

**Affiliations:** ^1^Programa de Pós-graduação, Universidade Ceuma, São LuísMA, Brazil; ^2^Programa de Pós-graduação, Universidade Federal do Maranhão, São LuísMA, Brazil; ^3^Centro de Especialidades Médicas VinhaisSão Luís, Brazil; ^4^Instituto Florence, São LuísMA, Brazil; ^5^Centro de Inovação e Ensaios Pré-Clínicos-CIEnP, FlorianópolisSC, Brazil; ^6^Vascular Biology and Inflammation Section, BHF Cardiovascular Centre of Excellence, King’s College LondonLondon, UK

**Keywords:** TRPA1, peripheral blood leukocytes, rheumatoid arthritis, polymorphonuclear cells, CD14^+^ cell activation

## Abstract

Patients with rheumatoid arthritis (RA) suffer from pain and joint disability. The transient receptor potential ankyrin 1 (TRPA1) channel expressed on sensory neurones and non-neuronal cells mediates pain transduction and inflammation and it has been implicated in RA. However, there is little information on the contribution of TRPA1 for human disease. Here, we investigated the expression of TRPA1 on peripheral blood leukocytes and the circulating levels of its endogenous activators 4-hydroxynonenal (4-HNE) and hydrogen peroxide (H_2_O_2_) in RA patients treated or not with the anti-rheumatic leflunomide (LFN) or the anti-TNFα adalimumab (ADA). We also assessed whether TRPA1 expression correlates with joint pain and disability, in addition to the immune changes in RA. TRPA1 expression on peripheral blood leukocytes correlated with pain severity and disability. TRPA1 levels on these cells were associated with the numbers of polymorphonuclear and the activation of CD14^+^ cells. No correlations were found between the lymphocyte population and TRPA1 expression, pain or disability. Patients recently diagnosed with RA expressed increased levels of TRPA1 on their leukocytes whilst treatment with either LFN or ADA down-regulated this receptor probably by reducing the numbers of polymorphonuclears and the activation of CD14^+^ cells. We suggest that the activation levels of CD14^+^ cells, the numbers of PMNs in the peripheral blood and the expression of TRPA1 on peripheral blood leukocytes correlate with RA progression, affecting joint pain sensitivity and loss of function.

## Introduction

Rheumatoid arthritis (RA) is a chronic inflammatory disease that affects approximately 1% of the population worldwide. Pain and loss of joint function are hallmarks of RA. It leads to diminished quality of life and increased burden on national health systems. Whilst non-steroidal and steroidal drugs have been used for pain management, RA treatment consists of disease-modifying anti-rheumatic drugs (DMARDs) and biologicals which do not always halt disease progression.

The transient receptor potential ankyrin 1 (TRPA1) is a member of the transient receptor potential (TRP) family, expressed on sensory neurones, in additional to non-neuronal cells (for review see: Fernandes et al., 2012; Chen and Hackos, 2015). Activated by endogenously produced inflammatory mediators such as the oxidative stress products 4-hydroxynonenal (4-HNE; Trevisani et al., 2007) and hydrogen peroxide (H_2_O_2_; Andersson et al., 2008; Bessac et al., 2008), TRPA1 has been implicated in pain transduction and inflammation (for review see: Fernandes et al., 2012; Chen and Hackos, 2015). Since the discovery of TRPA1, evidence has suggested that its functional expression on sensory neurones innervating the joints and non-neuronal cells composing the joints such as synoviocytes (Kochukov et al., 2006) and chondrocytes (Nummenmaa et al., 2016) may contribute to RA progression and the associated pain. Recently, functional TRPA1 was also found on immune cells (Billeter et al., 2015; Bertin et al., 2016; Mendes et al., 2016) that are known to play a role in RA. It is possible thus, that TRPA1 expression and activation on these cells further amplify inflammation as they migrate in to the joints during reactive RA. Although compelling, most of the evidence arises from animal models, with little known role in human disease, especially on how TRPA1 expression influences components of the immune response in RA.

Transient receptor potential ankyrin 1 expression on peripheral blood leukocytes has been linked to pain sensitivity in neuropathic patients (Sukenaga et al., 2016). We hypothesized that TRPA1 is up-regulated on peripheral blood leukocytes in RA and that this is associated with increased joint pain and reduced life quality. Therefore, we investigated the expression of TRPA1 on peripheral blood leukocytes and the circulating levels of 4-HNE and H_2_O_2_ in RA patients treated or not with either the DMARD leflunomide (LFN) or the anti-TNFα adalimumab (ADA). We also assessed whether TRPA1 expression correlates with joint pain and disability, in addition to the immune changes in RA.

## Materials and Methods

### Patients

A total of 40 patients (men and women) aged ≥ 30 years and clinically diagnosed with RA, were recruited for participation in the study. Patients included those who have just been diagnosed with RA but naïve for DMARDs and biologicals (*n* = 10); patients under LFN (20 mg/day, *per os. n* = 15) and patients under biological (ADA; 40 mg every 2 weeks, subcutaneously, *n* = 15) therapy for at least 8-months and less than 12-months. Patients presented a score ≥ 6 on the 2010 ACR-EULAR Classification Criteria For RA as previously detailed (Aletaha et al., 2010). Healthy subjects (*n* = 15) were used as controls and included those who had no history of recent infections, malignancy or other autoimmune diseases and no present or previous use of DMARDs, biologicals or experimental drugs. All individuals were assessed for pain level and disability by the Stanford Health Assessment Questionnaire (HAQ; for review see: Bruce and Fries, 2003). Accordingly, disability was evaluated by the HAQ-disability index which assesses the patient’s difficulty in performing his/her usual activities. Pain levels were determined through the visual analog scale (VAS), in which the patient indicates in a scale from 0 to 100, how much pain he/she felt in the past week (0- indicates no pain and 100-indicates severe pain). Ten millilitres (ml) of peripheral blood were collected from each patient for separation of plasma, serum and peripheral blood leukocytes. The study was reviewed and approved by the Human Research Ethics Committee of the Universidade CEUMA and was performed in compliance with the Declaration of Helsinki. A written informed consent was obtained from each participant.

### Serum Rheumatoid Factor

For rheumatoid factor quantification we used a rheumatoid factor particle-enhanced immunoturbidimetric method (RF II- Tina quant RF II, COBAS) in a COBAS INTEGRA 400 analyzer (Roche Diagnostics). For this, samples were initially diluted (1:5) and then, serial dilutions were prepared automatically (up to 1:128). Samples (50 μl) were incubated with latex particles coated with monoclonal anti-rheumatoid factor antibodies. Agglutination, denoted by formation of aggregates in positive samples, was determined turbidimetrically. Results are expressed as international units (IU) per litre (l) of sample.

### Serum C-Reactive Protein

For C-reactive protein quantification we used a CRP particle-enhanced immunoturbidimetric method (CRPL3- C Reactive Protein Gen 3, COBAS) in a COBAS INTEGRA 400 analyzer (Roche Diagnostics). For this, samples were initially diluted (1:2) and then, serial dilutions were prepared automatically (up to 1:128). Samples (50 μl) were incubated with latex particles coated with monoclonal anti-C-reactive protein antibodies. Agglutination, denoted by formation of aggregates in positive samples, was determined turbidimetrically. Results are expressed as milligrams (mg) per millilitre (ml) of sample.

### Plasma 4-HNE Levels

Plasma samples were separated by centrifugation (15 min, 800 × *g*) and evaluated for 4-HNE content by using a commercial OxiSelect^TM^ HNE Adduct Competitive ELISA Kit (Cell Biolabs, San Diego, CA, USA), according to the manufacturer’s instructions. Results are expressed as levels of 4-HNE in micrograms (μg) per ml.

### Plasma H_2_O_2_ Levels

H_2_O_2_ production in plasma samples was measured by using a H_2_O_2_/peroxidase assay kit (Amplex Red H_2_O_2_/Peroxidase assay kit; Molecular Probes, Invitrogen, Brazil). The assay was performed according to the manufacturer’s instructions. Briefly, 50 μl of plasma were incubated with 50 μl of a solution containing NaPO_4_ 0.05 M (pH 7.4), HRP 0.2 U/ml and Amplex Red Reagent (10-acetyl-3,7-dihydroxyphenoxazine) 25.7 mg/ml, for 2 h, at 37°C. Samples incubated with NaPO_4_ 0.05 M only were used as controls. After incubation, the reaction was read at 560 nm. After subtraction of background readings, the absorbance in each sample was compared with that obtained from a H_2_O_2_ (0–40 μM) standard curve. Results are expressed as H_2_O_2_ levels in micromolar (μM).

### Plasma TNFα Levels

The plasma levels of TNFα were evaluated by using a human cytometric bead array (CBA) cytokine kit (BD Biosciences, Brazil) according to the manufacturer’s instructions. Analysis was performed on a Facscalibur cytometer flow cytometer (BD Biosciences-Immunocytometry Systems). Results were calculated in CBA FCAP Array software (BD Biosciences, Brazil) and are expressed as pg/ml.

### TRPA1 Expression on Peripheral Blood Leukocytes

For analysis of human TRPA1 expression on peripheral blood leukocytes, samples were prepared and assayed in a commercial enzyme-linked immunosorbent assay kit, according to the manufacturer’s instructions (Cloud-Clone Corp, Houston, TX, USA). Briefly, samples were collected and the red blood cells were lysed with Cell Lysis Buffer (BD Pharmingen, Brazil). Total leukocytes were then separated by centrifugation (30 min, 800 × *g*), resuspended in ice-cold phosphate-buffered saline (PBS) and ultrasonicated for four times. Cell lysates were centrifuged (10 min, 800 × *g*, 4°C) to remove cell debris and kept at -70°C for further analysis. On the day of the experiments, samples were defrosted and assayed. Results are expressed as nanograms of TRPA1 per milligram of protein (ng/mg) in each sample.

### Flow Cytometry Analysis

For flow cytometry analysis, peripheral blood samples underwent red blood cell lysis as previously described for the quantification of TRPA1 on leukocytes. Single-cell suspensions were prepared and cells were then stained with Trypan blue (Sigma–Aldrich, Brazil) and assessed for viability in a haemocytometer. Cells (5 × 10^6^) were washed, resuspended in flow cytometry buffer [(2% fetal calf serum (Invitrogen, Brazil) in phosphate buffered saline-PBS (Sigma–Aldrich, Brazil)], and stained with directly conjugated monoclonal antibodies (BD Biosciences or eBiosciences, Brazil): anti-CD4 PE-Cy5 (clone RPA-T4), anti-CD14 FITC (clone 61D3), anti-CD19 FITC (clone HIB19), anti-CD25 PE (clone BC96; activation marker), anti-CD69 (clone FN50; activation marker), anti-CD127 FITC (clone eBioRDR5; activation marker), anti-HLA-DR PE-Cy5 (clone LN3). In order to discriminate regulatory T cells from activated CD4^+^ T cells, gates were placed on CD4^+^CD25^+^CD127^low^ and CD4^+^CD25^+^CD127^high^ cell populations, respectively. Events were acquired on a BD Accuri C6 (BD Biosciences-Immunocytometry Systems) and analyzed using FlowJo software (Tree Star Inc.). Additionally, differential cell populations [polymorphonuclear (PMNs) and mononuclear cells] were identified by size and granularity through flow cytometry. Results are expressed as well as number of cells per mm^3^, except for HLA-DR, expressed as mean fluorescence.

### Data Analysis

Data are represented as mean ± SD or median and interquartile range (25–75th; IQR), depending on their distribution. Accordingly, we used parametric (ANOVA followed by Bonferroni’s test) or non-parametric (Kruskal–Wallis) tests to determine the significance of differences between groups in the HAQ-DI and VAS pain scale scores; TRPA1, 4-HNE, H_2_O_2_, and TNFα levels; and leukocyte subpopulations. Correlations between the different parameters were determined using Spearman’s rho. Statistical analysis was undertaken using IBM SPSS Statistics 20. *p*-values < 0.05 were considered statistically significant.

## Results

### Subject Characteristics

Data depicted on **Table 1** shows that the arthritic population primarily consisted of women (75%). Also, most of the recently diagnosed arthritic patients (those receiving no specific treatment with anti-rheumatic drugs, NST group) presented disease for less than 10 years (80%) whilst 60% of the patients under treatment with LFN and 80% of the patients under treatment with ADA had disease symptoms for longer than 10 years. Swollen joints were present in 90% of the recently diagnosed patients. These patients also exhibited higher levels of C-reactive protein and rheumatoid factor. This symptom was less noticeable in those taking either LFN (33.3% of the patients) or ADA (20% of the patients). The incidence of lumps and deformities was higher in patients receiving LFN or ADA, in comparison with patients recently diagnosed with RA. Nine women and six men composed the population of healthy subjects, with an average age of 39.6 ± 4.6 years.

**Table 1:** Characteristics of the patients.

Variable	Arthritic patients		
	NST	LFN	ADA
Gender (*n*° of patients/%)	*n*° (%)	*n*° (%)	*n*° (%)
Age [Mean (SD)]	40.5 (8.39)	54.60 (12.07)	54.87 (10.88)
Male	1 (10%)	04 (26.7)	05 (33.3)
Female	09 (90%)	11 (73.3)	10 (66.7)
Duration of disease (in years)	*n*° (%)	*n*° (%)	*n*° (%)
<10	08 (80%)	06 (40%)	03 (20%)
10–19	02 (20%)	07 (46.7%)	05 (33.3%)
20–29	00 (0%)	02 (13.3%)	05 (33.3%)
>30	00 (0%)	00 (0%)	02 (13.4%)
Presence of swollen joints	*n*° (%)	*n*° (%)	*n* (%)
Yes	09 (90%)	05 (33.3%)	03 (20%)
No	01 (10%)	10 (66.7%)	12 (80%)
Presence of lumps or deformities	*n*° (%)	*n*° (%)	*n*° (%)
Yes	02 (20%)	07 (46.7%)	12 (80%)
No	08 (80%)	08 (53.3%)	03 (20%)
C-reactive protein, mg/l [Median (IQR)]	[31.0 (11.8-78.8)]	[1.1 (0.3-2.2)]	[1.3 (0.1-2.6)]
Rheumatoid factor, IU/ml [Median (IQR)	[160 (93.5-266.0)]	[31 (9.3-96.0)]	[20.0 (5.0-76.4)]

*Continuous variables are expressed as median (25–75th percentile; IQR). Categorical variables are summarized as *n* (%).*

### TRPA1 Expression on Leukocytes Correlates with Joint Pain and Disability

**FIGURE 1:**
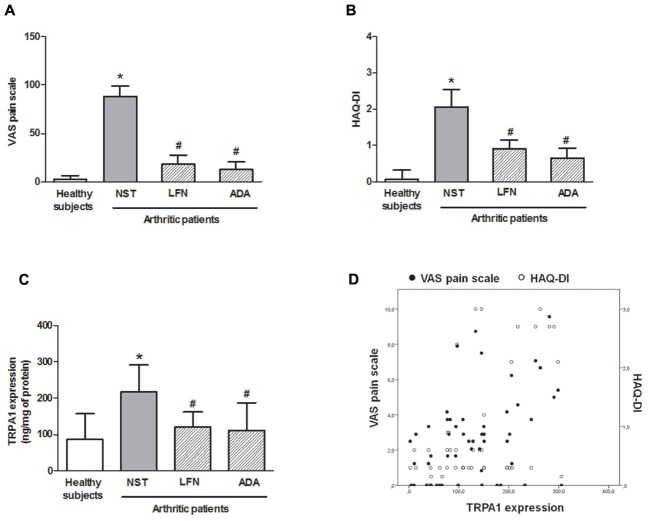
**(A)** Visual analog scale (VAS) pain scale, **(B)** HAQ-DI, and **(C)** TRPA1 expression on peripheral blood leukocytes in patients with rheumatoid arthritis (RA) treated or not with either leflunomide (LFN; *n* = 15) or adalimumab (ADA; *n* = 15), in comparison with patients recently diagnosed with RA but not yet receiving specific treatment with anti-rheumatic drugs (NST group; *n* = 10) and healthy subjects (*n* = 15). Data are expressed as mean ± SD. **(D)** Scatter plot for TRPA1 correlation with VAS pain scale and HAQ-DI. ^∗^*p* < 0.05, differs from healthy subjects; ^#^*p* < 0.05, differs from the NST group.

As shown in **Figure 1**, the NST group presented higher levels of joint pain (VAS pain scale; **Figure 1A**) and increased disability (HAQ disability index, HAQ-DI; **Figure 1B**) when compared with healthy subjects and patients treated with either LFN or ADA. Pain and disability levels correlated with TRPA1 expression levels on leukocytes (*r* = 0.329 and *r* = 0.390; respectively; *p* < 0.05; **Figure 1D**) as this receptor was markedly increased (2.6-fold) in patients who had been recently diagnosed with RA in comparison with those of healthy subjects and patients under LFN or ADA therapy (**Figure 1C**). Healthy subjects and patients taking LFN and ADA expressed ∼50% less TRPA1 on their leukocytes than those of NST patients.

### Plasma 4-HNE, H_2_O_2_, and TNFα Levels

**FIGURE 2:**
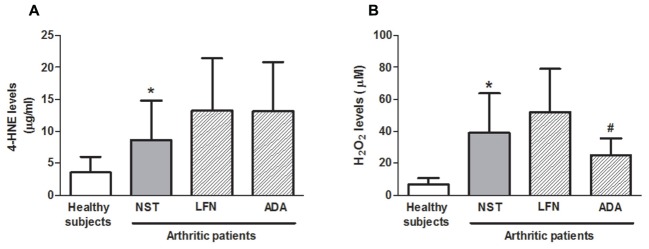
**Systemic** **(A)** 4-HNE and **(B)** H_2_O_2_ levels in patients with RA treated or not with either leflunomide (LFN; *n* = 15) or adalimumab (ADA; *n* = 15), in comparison with patients recently diagnosed with RA but not yet receiving specific treatment with anti-rheumatic drugs (NST group; *n* = 10) and healthy subjects (*n* = 15). Data are expressed as mean ± SD. ^∗^*p* < 0.05, differs from healthy subjects; ^#^*p* < 0.05, differs from the NST group.

4-HNE and H_2_O_2_ levels were increased by 2.4- and 5.6-fold in the NST group, respectively, in comparison with healthy subjects (**Figures 2A,B**; *p* < 0.05). On the other hand, 4-HNE levels did not differ between the arthritic groups (**Figure 2A**). Analysis of H_2_O_2_ showed that patients under ADA but not LFN treatment exhibited lower levels of this inflammatory mediator in their plasma (35.9 ± 27.0%) in comparison with those of the NST group (**Figure 2B**; *p* < 0.05). A correlation between the circulating levels of 4-HNE and disability in the NST group (*r* = 0.926; *p* < 0.05) was observed. Also, reduced joint pain and disability were accompanied by reduced levels of H_2_O_2_ in patients taking ADA (**Figure 2B**). On the other hand, not all samples presented detectable levels of TNFα. This cytokine was detected in only 5 out of 10 NST patients, 13 out of 15 patients taking LFN, 10 out of 15 patients taking ADA and 11 out of 15 healthy subjects. No differences were observed between the evaluated groups. Median and IQR values observed for TNFα are as follows: healthy subject group: 1.54 (0.98–1.66), NST group: 0.92 (0.66–4.82), RA patients under LFN treatment: 1.5 (0.63–5.37) and RA patients under ADA treatment: 0.88 (0.37–1.25). No correlations were found between TNFα levels and pain or disability, and neither between this cytokine and TRPA1 expression on whole peripheral blood leukocytes or the phenotypic characteristics of leukocytes.

### Correlation between the Number of PMNs and the Activation of CD14^+^ Cells with TRPA1 Expression

**FIGURE 3:**
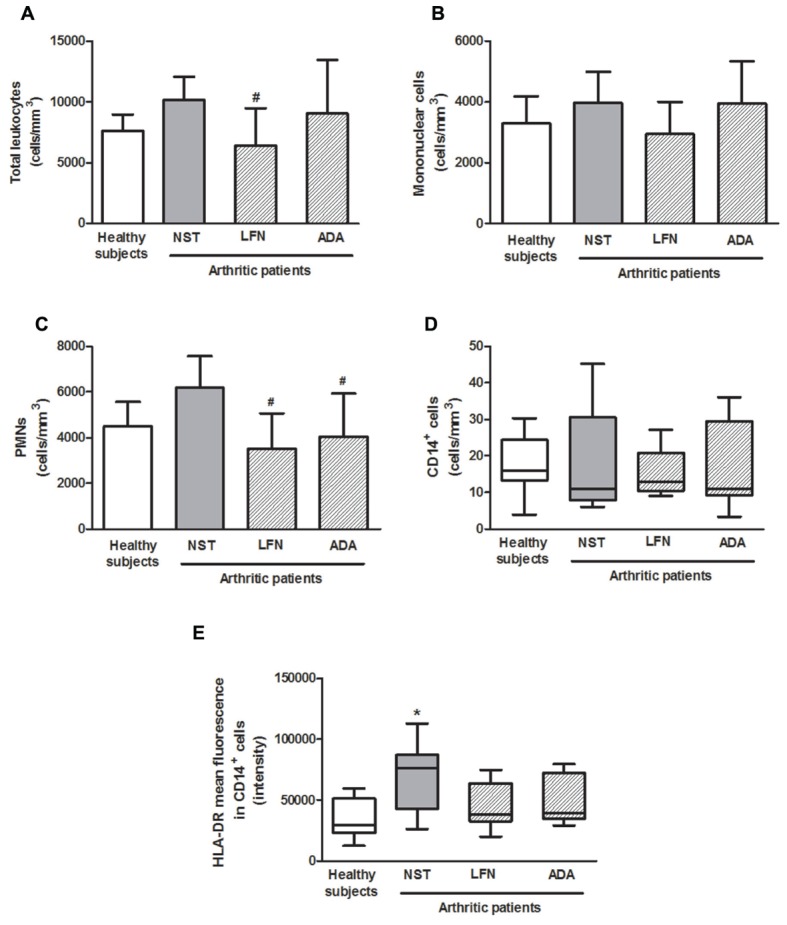
**Peripheral blood total** **(A)**, mononuclear **(B)**, and polymorphonuclear (PMNs) **(C)** cells; CD14^+^ cells **(D)** and HLA-DR mean fluorescence intensity in CD14^+^ cells **(E)** in patients with RA treated or not with either leflunomide (LFN; *n* = 15) or adalimumab (ADA; *n* = 15), in comparison with patients recently diagnosed with RA but not yet receiving specific treatment with anti-rheumatic drugs (NST group; *n* = 10) and healthy subjects (*n* = 15). Data are expressed as mean ± SD or median (25–75th percentile). ^∗^*p* < 0.05, differs from healthy subjects; ^#^*p* < 0.05, differs from the NST group.

The numbers of total leukocytes, mononuclear and PMN cells were analyzed. Total leukocyte and PMN numbers (**Figures 3A–C**) were markedly reduced in LFN patients (36.8 ± 30.1 and 43.1 ± 24.9%, respectively) in comparison with the NST group. Also, PMN numbers were diminished (34.8 ± 30.8%) in ADA patients (**Figure 3C**). No differences were observed in the mononuclear cell numbers between the groups (**Figure 2B**). Analysis of CD14^+^ cells showed that their numbers do not differ between the groups (**Figure 3D**). The activation of CD14^+^ cells, denoted by HLA-DR mean fluorescence, was significantly increased (1.9–2.6-fold) in samples obtained from NST patients in comparison to those of the other arthritic groups and healthy subjects (**Figure 3E**). Additionally, a correlation between the numbers of PMNs and TRPA1 expression (*r* = 0.476; *p* < 0.05); and pain (*r* = 0.349; *p* < 0.05) was observed.

Evaluation of lymphocyte subpopulations (**Table 2**) demonstrated an increase in the numbers of circulating CD4^+^ (T helper lymphocytes) CD8^+^ (T cytotoxic lymphocytes) and CD19^+^ (B lymphocytes) cells including those activated (CD25^+^, CD25^+^CD127^high^ or CD69^+^) in the NST group comparison with healthy subjects, however, this was not significant. LFN diminished the numbers of CD4^+^, CD4^+^CD25^+^CD127^low^ and CD8^+^ cells (reduction of 55.6 ± 21.6%, 55.0 ± 27.0, and 57.4 ± 28.8%, respectively). The same patients presented with 52.9% less circulating CD4^+^CD25^+^CD127^high^ cells than the NST group. No differences were observed for the other parameters between the tested groups.

**Table 2:** Peripheral blood leukocyte populations in healthy subjects and arthritic patients.

Leukocyte population (cells/mm^3^)	Healthy subjects	Arthritic patients		
		
		NST	LFN	ADA
CD4^+^	309.9 ± 192.6	420.0 ± 326.2	186.5 ± 90.7**^#^**	501.0 ± 349.2
CD4^+^CD25^+^CD127^high^	32.7 (21.3–51.8)	42.0 (27.5–132.6)	19.8 (13.2–24.3)**^#^**	47.8 (16.7–100.5)
CD4^+^CD25^+^CD127^low^	33.6 ± 16.1	43.3 ± 30.5	19.5 ± 11.7**^#^**	51.2 ± 35.1
CD4^+^CD69^+^	51.3 ± 33.0	93.8 ± 104.7	36.7 ± 24.6	75.8 ± 58.5
CD8^+^	138.6 ± 63.3	254.9 ± 276.8	108.6 ± 73.3**^#^**	272.0 ± 237.4
CD8^+^CD69^+^	23.0 ± 16.8	38.9 ± 33.8	17.1 ± 16.1	44.0 ± 47.3
CD19^+^	33.6 (20.4–45.5)	60.2 (17.0–101.1)	38.3 (19.8–75.7)	103.4 (22.9–148.7)
CD19^+^CD69^+^	8.5 ± 5.4	28.2 ± 34.8	17.5 ± 14.2	17.5 ± 19.2


*Samples were obtained from patients with rheumatoid arthritis (RA) treated or not with either leflunomide (LFN; *n* = 15) or adalimumab (ADA; *n* = 15) and compared with patients recently diagnosed with RA but not yet receiving specific treatment with anti-rheumatic drugs (NST group; *n* = 10) and healthy subjects (*n* = 15). Data are expressed as mean ± SD. ^#^*p* < 0.05, differs from the NST group. Variables are expressed as mean ± SD or median (25–75th percentile; IQR).*

No significant correlations were observed between TRPA1 expression and lymphocyte numbers and/or activation.

## Discussion

### TRPA1 Expression on Peripheral Blood Leukocytes Is Increased in RA Patients

Here, we show for the first time that TRPA1 protein expression on peripheral blood leukocytes positively correlates with joint pain and disability in RA patients. To the best of our knowledge, this is the first study showing that patients recently diagnosed with RA express increased levels of TRPA1 on their leukocytes and that the treatment with either LFN or ADA down-regulates this receptor.

Transient receptor potential ankyrin 1 channels expressed on neuronal tissues have been widely predicted as important transducers of pain sensation (for review see: Chen and Hackos, 2015). Recently, TRPA1 was found to contribute to joint pain and inflammation in a murine model of chronic arthritis induced by complete Freund’s adjuvant (Fernandes et al., 2011; Horváth et al., 2016). The discovery of functional TRPA1 on cells located in the joints such as synoviocytes and chondrocytes (Kochukov et al., 2006; Nummenmaa et al., 2016), in addition to its expression on immune cells (Billeter et al., 2015; Bertin et al., 2016; Mendes et al., 2016) have unveiled novel pathways on the peripheral modulation of pain. In a recent report, TRPA1 expression on peripheral blood leukocytes was found to be associated with pain sensitivity (Sukenaga et al., 2016). Indeed, patients with increased neuropathic pain symptoms presented with lower TRPA1 mRNA levels on their leukocytes. Similarly, Bell et al. (2014) showed that individuals with lower pain thresholds express lower TRPA1 mRNA levels in peripheral tissues such as the skin. Here, we show evidence on that TRPA1 protein expression is augmented in RA patients with increased pain and disability. Although both neuropathic and arthritic pain are of chronic nature, the different results obtained in these studies may be due to differences in TRPA1 quantification (mRNA × protein), length of disease and immunological components underlying these pathologies.

Here, TRPA1 expression was analyzed by an enzyme-linked immunosorbent assay. Enzyme-linked immunosorbent assays allow the simultaneous evaluation of many samples to a given protein with high sensitivity, but their specificity depends on assay conditions. Of note, their specificity may be comparable to those of western blot analysis for the detection of protein expression; however, results can differ depending on the antigenic preparation used in the two assays (Dittadi et al., 1993; Révélen et al., 2002; Liu et al., 2009). Importantly, TRPA1 expression was previously shown to be increased on leukocytes such as monocytes and lymphocytes in other inflammatory conditions including in LPS challenge and inflammatory bowel disease (Billeter et al., 2015; Bertin et al., 2016). It is possible that once increased, TRPA1 expression contributes to the progression or outcome of inflammatory diseases.

### 4-HNE and H_2_O_2_ Levels Are Increased in RA Patients

The endogenously produced oxidants 4-HNE and H_2_O_2_ are suggested to play a role in RA, contributing to disease progression (Remans et al., 2005; Yin et al., 2015). Recently, H_2_O_2_ was suggested to be critical to T cell differentiation (Abimannan et al., 2016). Indeed, the circulating levels of reactive oxygen species such as H_2_O_2_ and superoxide were recently found to be strongly and positively correlated with RA symptoms and disease activity markers (Khojah et al., 2016). Similarly, plasma 4-HNE levels are elevated in RA patients (Łuczaj et al., 2016). Both 4-HNE and H_2_O_2_ have been suggested as biomarkers for monitoring RA progression and are known activators of TRPA1 (Trevisani et al., 2007; Andersson et al., 2008; Bessac et al., 2008).

Here, 4-HNE levels were raised in RA. No significant differences were found in the 4-HNE levels between the arthritic groups, although a correlation between the circulating levels of 4-HNE and disability was observed in the NST group only. Our data also show that arthritic patients presented higher levels of H_2_O_2_ in their plasma, and that this oxidant is reduced in RA patients under ADA therapy.

Neither 4-HNE nor H_2_O_2_ were found to correlate with the TRPA1 expression on peripheral blood leukocytes or pain. It is possible that the ongoing oxidative stress may not affect TRPA1 expression on these cells, and thus, may not affect pain sensitivity mediated by this channel in RA.

TNFα plays a central role in RA pathophysiology, mediating bone resorption and joint pain and inflammation in RA, in addition to contributing to extra-articular disease (for review see: McInnes et al., 2016). Recent evidence indicates that TRPA1 mediates TNFα-induced pain *in vivo* (Fernandes et al., 2011), and that this cytokine enhances TRPA1 expression on non-neuronal cells (El Karim et al., 2015). Therefore, we assessed the circulating levels of TNFα in healthy subjects and in RA patients. No differences were observed between the groups. Of note, we were not able to detect circulating TNFα in 50% of the samples obtained from NST patients. Although being important for the transition of RA toward chronicity, TNFα levels may peak during the maintenance of established disease (McInnes et al., 2016). TNFα levels were not correlated with any of the parameters assessed. This may be related to the fact this cytokine was not detected in all patients.

### The Correlation between TRPA1 Expression on Peripheral Blood Leukocytes and Pain and Disability in RA Is Associated with the Numbers of PMN and Activation of CD14^+^ Cells

Our data shows that the correlation between TRPA1 expression on peripheral blood leukocytes, pain and disability in arthritic patients is associated with the numbers of PMNs and with the activation of CD14^+^ cells. Horváth et al. (2016) found that TRPA1 ablation decreases neutrophil accumulation into the joints of animals with arthritis. Also, TRPA1 was recently shown to mediate the acute inflammatory responses mediated by CD14^+^ expressing cells such as macrophages (Mendes et al., 2016) and monocytes (Billeter et al., 2015). Additionally, the functional expression of TRPA1 on CD4^+^ lymphocytes was previously reported and the activation of this channel was suggested to down-regulate T-cell mediated responses in chronic inflammation (Bertin et al., 2016). However, we did not observe any correlations between the circulating lymphocyte subpopulations and TRPA1 expression or pain and disability in arthritic patients. Also, no correlations were found between lymphocyte subpopulations and the systemic levels of 4-HNE and H_2_O_2_. It is possible though that lymphocytes located in to the joints differently influence RA progression as this receptor may suffer the influence of a plethora of inflammatory molecules (i.e., oxidative stress products and cytokines such as TNFα) released in higher levels in this microenvironment.

We found that RA patients receiving either LFN or ADA presented lower numbers of circulating PMNs and less activation of CD14^+^ cells than those of the NST group. Both LFN and ADA are able to directly suppress immune cell proliferation and migration in to the joints. LFN is known to block T cell proliferation by inhibiting dihydro-orotate dehydrogenase and the synthesis of pyrimidine (Fragoso and Brooks, 2015), and to reduce the expression of adhesion molecules on peripheral blood mononuclear cells and their migration in to the inflamed synovia (Grisar et al., 2004). ADA was also shown to reduce peripheral blood leukocyte (monocytes and PMNs) adhesion to the endothelium (Ríos-Navarro et al., 2015). Inhibitory effects on T cell proliferation via macrophage-dependent mechanisms were also observed for ADA (Vos et al., 2011). Taking these evidences in to account, it is possible to suggest that in the NST group, infiltrating leukocytes expressing TRPA1 may contribute to exacerbate joint inflammation, a response that may be attenuated in patients receiving anti-rheumatic therapy.

In summary, our data demonstrates that TRPA1 expression on peripheral blood leukocytes is increased in NST RA patients and this is associated with higher numbers of circulating neutrophils and increased CD14^+^ cell activation. In turn, these changes affect pain and disability as TRPA1-expressing leukocytes may migrate in to the joints during reactive RA further amplifying inflammation. Finally, reduced TRPA1 expression in patients treated with either LFN or ADA is accompanied by reduction of the circulating PMN population and decreased activation of CD14^+^ cells, thus resulting in decreased pain and disability in RA. These results suggest that the activation levels of CD14^+^ cells and the numbers of PMNs in the peripheral blood is associated with TRPA1 expression and this may impact RA progression.

## Ethics Statement

The study was reviewed and approved by the Human Research Ethics Committee of the Universidade CEUMA and was performed in compliance with the Declaration of Helsinki. A written informed consent was obtained from each participant.

## Author Contributions

IP, SM, DP, TM, VC, CM, MM, MG, VM-N, SGM, JC, SB, and EF contributed to conception, design, data acquisition, analysis, and interpretation, drafted and critically revised the manuscript. All authors gave final approval and agree to be accountable for all aspects of the work.

## Conflict of Interest Statement

The authors declare that the research was conducted in the absence of any commercial or financial relationships that could be construed as a potential conflict of interest.

## References

[B1] Abimannan T. Peroumal D. Parida J. R. Barik P. K. Padhan P. Devadas S. (2016). Oxidative stress modulates the cytokine response of differentiated Th17 and Th1 cells. *Free Radic. Biol. Med.* 99 352–363. 10.1016/j.freeradbiomed.2016.08.026 27567538

[B2] Aletaha D. Neogi T. Silman A. J. Funovits J. Felson D. T. Bingham C. O. I. I. I. (2010). Rheumatoid arthritis classification criteria: an American College of Rheumatology/European League Against Rheumatism collaborative initiative. *Arthritis Rheum.* 62 2569–2581. 10.1002/art.27584 20872595

[B3] Andersson D. A. Gentry C. Moss S. Bevan S. (2008). Transient receptor potential A1 is a sensory receptor for multiple products of oxidative stress. *J. Neurosci.* 28 2485–2494. 10.1523/JNEUROSCI.5369-07.2008 18322093 PMC2709206

[B4] Bell J. T. Loomis A. K. Butcher L. M. Gao F. Zhang B. Hyde C. L. (2014). Differential methylation of the TRPA1 promoter in pain sensitivity. *Nat. Commun.* 5 2978 10.1038/ncomms3978 PMC3926001 24496475

[B5] Bertin S. Aoki-Nonaka Y. Lee J. de Jong P. R. Kim P. Han T. (2016). The TRPA1 ion channel is expressed in CD4+ T cells and restrains T-cell-mediated colitis through inhibition of TRPV1. *Gut* 10.1136/gutjnl-2015-310710 [Epub ahead of print], PMC5173457 27325418

[B6] Bessac B. F. Sivula M. von Hehn C. A. Escalera J. Cohn L. Jordt S. E. (2008). TRPA1 is a major oxidant sensor in murine airway sensory neurons. *J. Clin. Invest.* 118 1899–1910. 10.1172/JCI34192 18398506 PMC2289796

[B7] Billeter A. T. Galbraith N. Walker S. Lawson C. Gardner S. A. Sarojini H. (2015). TRPA1 mediates the effects of hypothermia on the monocyte inflammatory response. *Surgery* 158 646–654. 10.1016/j.surg.2015.03.065 26054320

[B8] Bruce B. Fries J. F. (2003). The stanford health assessment questionnaire: dimensions and practical applications. *Health Qual. Life Outcomes* 1 20 10.1186/1477-7525-1-20 PMC165587 12831398

[B9] Chen J. Hackos D. H. (2015). TRPA1 as a drug target–promise and challenges. *Naunyn Schmiedebergs Arch. Pharmacol.* 388 451–463. 10.1007/s00210-015-1088-3 25640188 PMC4359712

[B10] Dittadi R. Catozzi L. Gion M. Brazzale A. Capitanio G. Gelli M. C. (1993). Comparison between western blotting, immunohistochemical and ELISA assay for p185neu quantitation in breast cancer specimens. *Anticancer Res.* 13 1821–1824. 7903522

[B11] El Karim I. McCrudden M. T. Linden G. J. Abdullah H. Curtis T. M. McGahon M. (2015). TNF-α-induced p38MAPK activation regulates TRPA1 and TRPV4 activity in odontoblast-like cells. *Am. J. Pathol.* 185 2994–3002. 10.1016/j.ajpath.2015.07.020 26358221

[B12] Fernandes E. S. Fernandes M. A. Keeble J. E. (2012). The functions of TRPA1 and TRPV1: moving away from sensory nerves. *Br. J. Pharmacol.* 166 510–521. 10.1111/j.1476-5381.2012.01851.x 22233379 PMC3417484

[B13] Fernandes E. S. Russell F. A. Spina D. McDougall J. J. Graepel R. Gentry C. (2011). A distinct role for transient receptor potential ankyrin 1, in addition to transient receptor potential vanilloid 1, in tumor necrosis factor α-induced inflammatory hyperalgesia and Freund’s complete adjuvant-induced monarthritis. *Arthritis Rheum.* 63 819–829. 10.1002/art.30150 21360511

[B14] Fragoso Y. D. Brooks J. B. (2015). Leflunomide and teriflunomide: altering the metabolism of pyrimidines for the treatment of autoimmune diseases. *Exp. Rev. Clin. Pharmacol.* 8 315–320. 10.1586/17512433.2015.1019343 25712857

[B15] Grisar J. Aringer M. Köller M. D. Stummvoll G. H. Eselböck D. Zwölfer B. (2004). Leflunomide inhibits transendothelial migration of peripheral blood mononuclear cells. *Ann. Rheum. Dis.* 63 1632–1637. 10.1136/ard.2003.018440 15547088 PMC1754829

[B16] Horváth Á. Tékus V. Boros M. Pozsgai G. Botz B. Borbély É (2016). Transient receptor potential ankyrin 1 (TRPA1) receptor is involved in chronic arthritis: in vivo study using TRPA1-deficient mice. *Arthritis Res. Ther.* 18 6 10.1186/s13075-015-0904-y PMC4718022 26746673

[B17] Khojah H. M. Ahmed S. Abdel-Rahman M. S. Hamza A. B. (2016). Reactive oxygen and nitrogen species in patients with rheumatoid arthritis as potential biomarkers for disease activity and the role of antioxidants. *Free Radic. Biol. Med.* 97 285–291. 10.1016/j.freeradbiomed.2016.06.020 27344969

[B18] Kochukov M. Y. McNearney T. A. Fu Y. Westlund K. N. (2006). Thermosensitive TRP ion channels mediate cytosolic calcium response in human synoviocytes. *Am. J. Physiol. Cell Physiol.* 291 C424–C432. 10.1152/ajpcell.00553.2005 16597917

[B19] Liu D. Schuster T. Baumann M. Roos M. Sollinger D. Lutz J. (2009). Comparison of immunoassays for the selective measurement of human high-molecular weight adiponectin. *Clin. Chem.* 55 568–572. 10.1373/clinchem.2008.112425 19168560

[B20] Łuczaj W. Gindzienska-Sieskiewicz E. Jarocka-Karpowicz I. Andrisic L. Sierakowski S. Zarkovic N. (2016). The onset of lipid peroxidation in rheumatoid arthritis: consequences and monitoring. *Free Radic. Res.* 50 304–313. 10.3109/10715762.2015.1112901 26764956

[B21] McInnes I. B. Buckley C. D. Isaacs J. D. (2016). Cytokines in rheumatoid arthritis - shaping the immunological landscape. *Nat. Rev. Rheumatol.* 12 63–68. 10.1038/nrrheum.2015.171 26656659

[B22] Mendes S. J. Sousa F. I. Pereira D. M. Ferro T. A. Pereira I. C. Silva B. L. (2016). Cinnamaldehyde modulates LPS-induced systemic inflammatory response syndrome through TRPA1-dependent and independent mechanisms. *Int. Immunopharmacol.* 34 60–70. 10.1016/j.intimp.2016.02.012 26922677

[B23] Nummenmaa E. Hämäläinen M. Moilanen L. J. Paukkeri E. L. Nieminen R. M. Moilanen T. (2016). Transient receptor potential ankyrin 1 (TRPA1) is functionally expressed in primary human osteoarthritic chondrocytes. *Arthritis Res. Ther.* 18 185 10.1186/s13075-016-1080-4 PMC4982008 27515912

[B24] Remans P. H. van Oosterhout M. Smeets T. J. Sanders M. Frederiks W. M. Reedquist K. A. (2005). Intracellular free radical production in synovial T lymphocytes from patients with rheumatoid arthritis. *Arthritis Rheum.* 52 2003–2009. 10.1002/art.21111 15986371

[B25] Révélen R. D’Arbonneau F. Guillevin L. Bordron A. Youinou P. Dueymes M. (2002). Comparison of cell-ELISA, flow cytometry and Western blotting for the detection of antiendothelial cell antibodies. *Clin. Exp. Rheumatol.* 20 19–26. 11892703

[B26] Ríos-Navarro C. de Pablo C. Collado-Diaz V. Orden S. Blas-Garcia A. Martínez-Cuesta M. Á (2015). Differential effects of anti-TNF-α and anti-IL-12/23 agents on human leukocyte-endothelial cell interactions. *Eur. J. Pharmacol.* 765 355–365. 10.1016/j.ejphar.2015.08.054 26344475

[B27] Sukenaga N. Ikeda-Miyagawa Y. Tanada D. Tunetoh T. Nakano S. Inui T. (2016). Correlation between DNA methylation of TRPA1 and chronic pain states in human whole blood cells. *Pain Med* 17 1906–1910. 10.1093/pm/pnv088 26849948

[B28] Trevisani M. Siemens J. Materazzi S. Bautista D. M. Nassini R. Campi B. (2007). 4-Hydroxynonenal, an endogenous aldehyde, causes pain and neurogenic inflammation through activation of the irritant receptor TRPA1. *Proc. Natl. Acad. Sci. U.S.A.* 104 13519–13524. 10.1073/pnas.0705923104 17684094 PMC1948902

[B29] Vos A. C. Wildenberg M. E. Duijvestein M. Verhaar A. P. van den Brink G. R. Hommes D. W. (2011). Anti-tumor necrosis factor-α antibodies induce regulatory macrophages in an Fc region-dependent manner. *Gastroenterology* 140 221–230. 10.1053/j.gastro.2010.10.008 20955706

[B30] Yin G. Wang Y. Cen X. M. Yang M. Liang Y. Xie Q. B. (2015). Lipid peroxidation-mediated inflammation promotes cell apoptosis through activation of NF-κB pathway in rheumatoid arthritis synovial cells. *Mediators Inflamm.* 2015 10 10.1155/2015/460310 PMC4337269 25741130

